# A near-infrared fluorescent aptananosensor enables selective detection of the stress hormone cortisol in artificial cerebrospinal fluid

**DOI:** 10.1039/d5sd00085h

**Published:** 2025-10-07

**Authors:** Jessica Kretli Zanetti, Maria Celina Stefoni, Catarina Ferraz, Amelia Ryan, Atara Israel, Ryan M. Williams

**Affiliations:** a Biomedical Engineering, The City College of New York New York NY 10031 USA ryan.williams@stonybrookmedicine.edu; b Departamento de Química Inorgánica, Analítica y Química Física, Facultad de Ciencias Exactas y Naturales (DQIAQF), Universidad de Buenos Aires, and Instituto de Química Física de los Materiales, Medio Ambiente y Energía (INQUIMAE), CONICET-UBA Buenos Aires C1428 Argentina; c Department of Medicine, Division of Nephrology & Hypertension, Stony Brook University Stony Brook NY 11794 USA

## Abstract

Cortisol is a hormone which regulates the body's response to stressors. Detection and monitoring of cortisol levels can provide information about physical and psychological health, thus it is essential to develop a sensor that can detect it in a sensitive manner. This study presents a biocompatible near-infrared fluorescent sensor, wherein single-walled carbon nanotubes (SWCNT) are functionalized with a cortisol-specific aptamer. We found this sensor was capable of detecting cortisol from 37.5 μg mL^−1^ to 300 μg mL^−1^ and that it was selective for cortisol compared to the similar molecule estrogen. Moreover, SWCNT functionalized with non-specific oligonucleotides did not exhibit a concentration-dependent response to cortisol, demonstrating the specificity provided by the aptamer sequence. The sensor also demonstrated the ability to detect cortisol in artificial cerebrospinal fluid. We anticipate that future optimization of this sensor will enable potential point-of-care or implantable device-based rapid detection of cortisol, with the potential for improving overall patient health and stress.

## Abstract 2

In this study, we used carbon nanotubes and a ssDNA aptamer to design a fluorescent sensor to detect cortisol, which we showed we could do in a selective manner compared to estradiol and could do in artificial cerebrospinal fluid.

## Graphical abstract

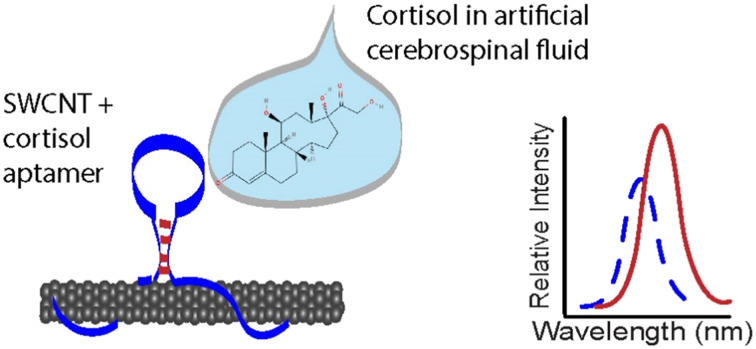

## Introduction

Cortisol is a steroid hormone that regulates essential physiological processes, including electrolyte balance, blood pressure, immune modulation, and metabolism.^1^ The level of this hormone in biofluids varies throughout the day, reaching its peak in the morning and its lowest level at night.^2^ Beyond its circadian rhythm, cortisol is secreted in response to stress, increasing blood pressure to provide metabolic energy from fat and glucose to muscles and brain. Prolonged exposure to stressors raises cortisol levels, thereby increasing the risk of cardiovascular diseases.^3^ Moreover, elevated cortisol levels are associated with reduced cognitive functioning, higher risk of dementia, and development of Alzheimer's disease.^4^ Thus, to support early diagnosis and intervention, it is imperative to monitor cortisol levels in a sensitive, specific, and cost-effective manner.

Several cortisol biosensors have been developed^5–10^ using numerous detection methods, such as electrochemistry,^11–21^ field-effects transistors (FETs),^22,23^ FRET^24^ and surface plasmon resonance (SPR).^25^ For instance, cortisol detection was achieved in sweat using a wearable sensing device that includes a microfluidic chip and a three-electrode system, where the working electrode was modified with mesoporous silica nanochannels.^26^ Another cortisol sensor developed^27^ utilized competitive lateral flow immunoassay (LFIA) for cortisol detection in saliva, utilizing anti-cortisol antibodies that are functionalized both on gold nanoparticles and in the sensor's detection zone. Although these works successfully detected cortisol, they are not well-suited for implantable sensors. Optical sensors based on single-walled carbon nanotubes (SWCNT) offer a promising alternative.

Single-walled carbon nanotubes can be visualized as a single sheet of graphene rolled into a cylinder, denoted by an (*n*, *m*) index, determined by the vector along which this sheet is rolled.^28^ SWCNT are inherently fluorescent in the tissue-transparent near infrared (NIR) region, and do not exhibit photobleaching, making them an ideal candidate for *in vitro* and *in vivo* applications.^29,30^ SWCNT can be functionalized with biomolecular recognition elements or may have inherent binding to analytes of interest. The interaction of an analyte with the functionalized SWCNT surfaced modulates the fluorescence of the SWCNT, causing solvatochromic or intensity-based responses.^29^ There have been several examples of DNA aptasensors incorporating SWCNT, wherein carbon nanotubes are functionalized with an aptamer, which in turn interacts with the target analyte, producing a detectable change in fluorescence.^31–37^ The aptamer can either wrap the SWCNT or be tethered to the SWCNT through a secondary DNA sequence.

For example, a serotonin sensor was developed by directly functionalizing a specific aptamer to SWCNT,^34^ which was capable of detecting serotonin release pattern from activated platelets with single-cell resolution. In contrast, a sensor that sucessfully detected unlabelled proteins from *Escherichia coli* and *Pichia pastoris* was developed with an aptamer-anchor polynucleotide sequence, using chemical spacers.^38^ Further, SWCNT-based sensors for human steroids were developed using Corona phase molecular recognition (CoPhMoRe). In that study, polymeric interactions with SWCNT were screened against various steroids, in turn selecting two sensors to detect cortisol and progesterne. The progesterone sensor in that study was further assessed for its *in vivo* sensor capabilities.^39^ Despite these advances, we sought to assess the selectivity and sensitivity of a SWCNT sensor using a biorecognition element.

In this work, we developed a cortisol aptananosensor based on SWCNT optical signal transduction. An aptamer with known affinity for cortisol^40^ was utilized to simultaneously disperse SWCNT in solution and to induce cortisol binding. This represents an advance in hormone detection with SWCNTs by introducing a specific biorecognition element that outperforms non-specific ssDNA-based constructs, as demonstrated in this work by comparisons with a random 40-mer sequence and (GT)_20_. We found that this sensor can report relative changes in concentrations of cortisol in solution and does so in a selective manner when challenged with estrogen, a steroid hormone of similar structure to cortisol. Finally, we demonstrated that the sensor is functional in artificial cerebrospinal fluid, portending its potential for further translation in disease diagnosis.

## Methods

### Aptananosensor synthesis and control constructs

High-pressure carbon monoxide (HiPCO) SWCNT (NanoIntegris Technologies, Boisbriand, Quebec) were suspended in solution separately with three oligonucleotide sequences (Table 1 and Below); Integrated DNA Technologies, Coralville, IA) in a 1 : 2 mass ratio with the addition of 1× PBS as previously described.^41^ The same was done when using (6,5) chirality enriched SWCNT (Sigma Aldrich).

**Table 1:** Oligonucleotide sequences used to suspend SWCNT

Cortisol Aptamer^40^	5′- ATGGGCAATGCGGGGTGGAGAATGGTTGCCGCACTTCGGC-3′
Random 40mer ssDNA	5′- AGCTGATCGTACGTTGCGATCAGTGGCTAACCGTGAATCC -3′
(GT)_20_	5′-GTGTGTGTGTGTGTGTGTGTGTGTGTGTGTGTGT-3′

#### Cortisol aptamer

The cortisol aptamer used in these experiments was previously published as a sequence that successfully detects cortisol, obtained through the SELEX process.^40^ This aptamer has been used in several applications, with a limit of detection of 1 ng mL^−1^ (2.7 nM)^42^ reported in an assay with gold nanoparticles.

#### Random 40mer

To verify the specificity and selectivity of the cortisol aptamer, a 40 base pairs ssDNA sequence was randomly generated to match the length of the cortisol aptamer with no discernable tertiary structure, G-quadruplex structures, or protein coding regions.

#### (GT)_20_

The (GT)_20_ sequence was selected since poly-GT sequences are known to suspend SWCNT well and are the same length as the cortisol aptamer.^43,44^ (GT)_20_ has no selective affinity towards cortisol and was therefore used as a control to evaluate whether the use of an aptamer provides specificity.

The sample was sonicated at 40% amplitude for 60 minutes in an ice bath with a 120 W ultrasonicator with 1/8′′ probe microtip (Fisher Scientific; Hampton, NH). The suspension was then ultra centrifuged at 58 000 × *g* for 1 hour (Beckman Coulter; California, USA) to remove impurities and aggregates. The top 75% of the supernatant was collected. Prior to use, the SWCNT suspensions were filtered through a 100 kDa centrifugal filter (Millipore Sigma, Burlington, MA) to remove excess unbound oligonucleotides, and resuspended in 100–200 μl of 1× PBS.

### Evaluation of sensor concentration and dispersion

SWCNT suspended by oligonucleotides were characterized with a V-730 UV-visible absorption spectrophotometer measured over 300–1100 nm (Jasco Inc., Easton, MD) using the molar extinction coefficient Abs_630_ = 0.036 L mg^−1^ cm^−1^ to determine the concentration of each as previously described.^45^

### Near-infrared fluorescence spectroscopy

Near-infrared fluorescence spectra of the aptananosensor and controls were acquired *via* a NIR plate reader (ClaIR, Photon, *etc.*, Montreal, Quebec) with laser source excitation wavelengths 655 nm and 730 nm. Near-infrared spectral acquisitions were performed in a Corning half-area UV 96-well plate (Fisher Scientific, Waltham, MA) with fluorescence spectra acquired between 900 and 1700 nm and excitation laser power set at 1750 mW at an exposure time of 500 ms.

### Functionality of the aptananosensor

Hydrocortisone 98% (Fisher Scientific, Waltham, MA) was solubilized in a 1% dimethyl sulfoxide (DMSO) solution (Fisher Scientific, Waltham, MA) in PBS. Varying concentrations of cortisol were added to 0.5 mg L^−1^ of sensor in 1x PBS for a total sample volume of 120 μL. A NIR spectral baseline was acquired as above before the addition of cortisol. Spectra were obtained every 15 minutes for 3 hours thereafter.

### Sensor specificity assessments

To evaluate sensors specificity to cortisol, the biologically and chemically similar hormone β-estradiol (Fisher Scientific, Waltham, MA) was evaluated at an equal concentration to cortisol. Fluorescence was measured every 15 minutes over 3 hours and analyzed for shifts in center wavelength.

### Sensor performance on different media

To evaluate sensor performance in artificial sweat (a-sweat) and artificial cerebrospinal fluid (aCSF) (both from Fisher Scientific, Waltham, MA), a 100 μL solution of 0.6 mg L^−1^ of SWCNT-aptamer was added to each well, in both a-sweat and aCSF. Then, 20 μL of 900 μg mL^−1^ of cortisol in 1% DMSO was added, yielding an 80% concentration of either a-sweat or aCSF in each well.

### Data analysis

All experiments were performed in triplicate. Samples which did not exhibit fluorescence, indicating poor or failed fluorescent detection, or non-physical (>20 nm) wavelength changes, indicating poor measurement or fit, were excluded. Individual SWCNT chiral emission peaks were identified according to published studies.^41,45^ Each peak was fit using a pseudo-Voigt model with a custom MATLAB code (code available upon request) and data were used for analyses when model fit *R*^2^ was greater than 0.95. Triplicate averages and mean standard deviations were obtained and reported. Intensity changes and center wavelength shifts were normalized by subtracting the sample response to SWCNT intensity/center wavelength in 1% DMSO in absence of any analyte. One-way ANOVA analysis was performed with Tukey posthoc analyses.

## Results and discussion

### The aptananosensor demonstrates sensitive concentration-dependent response to cortisol

**Fig. 1:**
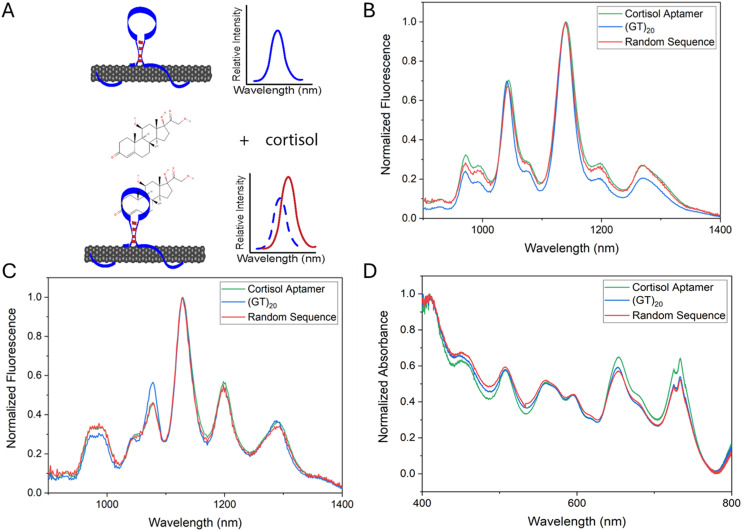
Cortisol detection strategy and sensor/control characterization. A) A SWCNT-based aptananosensor detection strategy for the stress hormone cortisol. Fluorescence spectra of SWCNT functionalized with cortisol aptamer, (GT)_20_ and a random 40mer sequence with laser excitation at (B) 655 nm and (C) 730 nm. (D) UV-vis absorbance spectra of the SWCNT constructs.

In order to engineer a fluorescent aptananosensor for the stress hormone cortisol, we non-covalently suspended the SWCNT in solution directly with a cortisol-selective aptamer (Fig. 1A). HiPCO SWCNTs were suspended well in solution with each of the cortisol aptamer, (GT)_20_ ssDNA, and random 40-mer ssDNA sequence. The resulting constructs were inherently fluorescent under red laser (655 nm and 730 nm) excitation (Fig. 1B and C). The UV-vis spectra of these (Fig. 1D) demonstrate that each of the 3 sequence-SWCNT constructs were suspended well in solution with multiple abundant SWCNT species present.

When dispersed with ssDNA, SWCNT are typically water soluble. However, cortisol is not water soluble, and thus it was necessary to select a solvent to solubilize cortisol while minimizing interference with the SWCNT fluorescence. To evaluate the influence of the solvent on sensors fluorescence, ethanol, 1% DMSO, and 0.1% DMSO, were added to the SWCNT-aptamer construct. The addition of 0.1% DMSO and 1% DMSO caused a center wavelength shift (Fig. S1A) comparable to the addition of PBS. However, SWCNT fluorescence in 0.1% DMSO behaved differently over time than PBS, exhibiting more pronounced center wavelength shifts (Fig. S1C). When analyzing the (7,6) peak, the behavior observed with 1% DMSO was also the most similar to that in PBS (Fig. S2). Therefore, we performed all further sensor analysis in 1% DMSO.

**Fig. 2:**
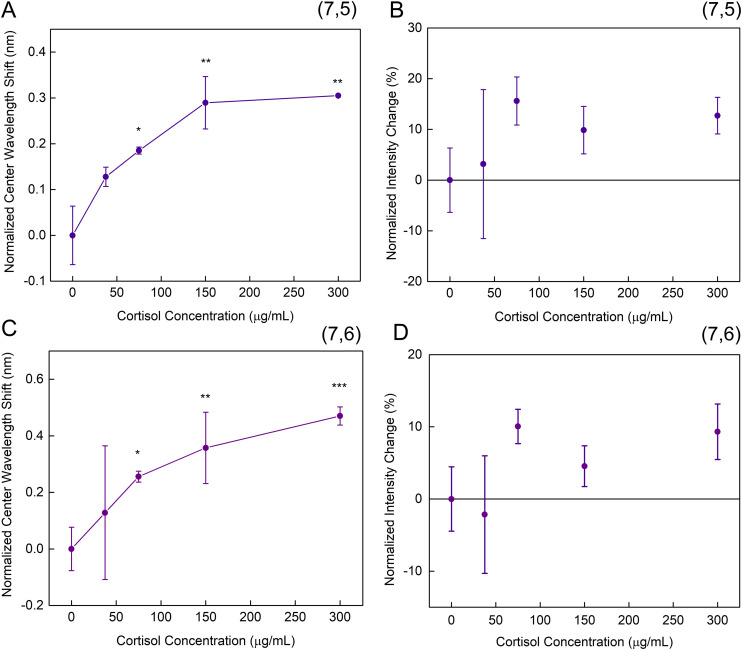
Dynamic range of aptananosensor response to cortisol. (A) Normalized center wavelength shift and (B) normalized intensity change, in (7,5) SWCNTs peak with increasing cortisol concentration after 3 hours. (C) Normalized center wavelength shift and (D) normalized intensity change, in (7,6) SWCNTs peak with increasing cortisol concentration after 3 hours. * = *p* < 0.5, ** = *p* < 0.01, *** = *p* < 0.001.

We then evaluated sensor response to increasing cortisol concentrations from 37.5 μg mL^−1^ to 300 μg mL^−1^. The concentration response was analyzed with (6,5)-enriched carbon nanotubes (Fig. S3). The (7,5) and (7,6) peaks exhibited a positive correlation between wavelength change and concentration, though neither did for intensity. The only significant difference to the absence of cortisol was obtained at 125 μg mL^−1^, hence, a concentration range was evaluated around this value with HiPCO SWCNTs. With aptamer functionalized HiPCO SWCNTs, the center wavelength shift of (7,5) and (7,6) peaks increased with higher cortisol concentrations, reaching a plateau around 300 μg mL^−1^ (Fig. 2A and C). Although both chiralities exhibit a monotonic response to cortisol and reach the plateau around the same concentration, the concentration curves are different. This could be attributed to the unique conformational structures that each SWCNT lattice induce in the aptamer, therefore, both chiralities were analyzed.^46–48^ Though shifts as small as 0.2–0.3 nm were observed, they were repeatable, with a small variability that significantly differed from controls. This response appeared to be concentration-dependent and consistent with a site-specific binding curve that saturated at higher concentrations. The intensity changes appeared to be somewhat binary (Fig. 2B and D), with detection was noted between 75–300 μg mL^−1^. The aptamer-SWCNT sensor presented in this work exhibited detection of cortisol from 37.5 μg mL^−1^ to 300 μg mL^−1^. As 37.5 μg mL^−1^ was the lowest concentration we evaluated, it did not approach the clinically relevant range in sweat from 0.008–0.141 μg mL^−1^.^49^ However, this is within the range of the prior study which performed CoPhMoRe-based SWCNT sensor development, as the cortisol sensor in that work operated linearly between ∼10–70 μg mL^−1^.^39^ Potential improvements in the detection range could be attained by using chirality-sorted SWCNTs to limit spectral overlap^50–52^ and by exploring other cortisol aptamers.^53–55^

### The aptananosensor induces specific response to cortisol while non-specific ssDNA sequences do not

**Fig. 3:**
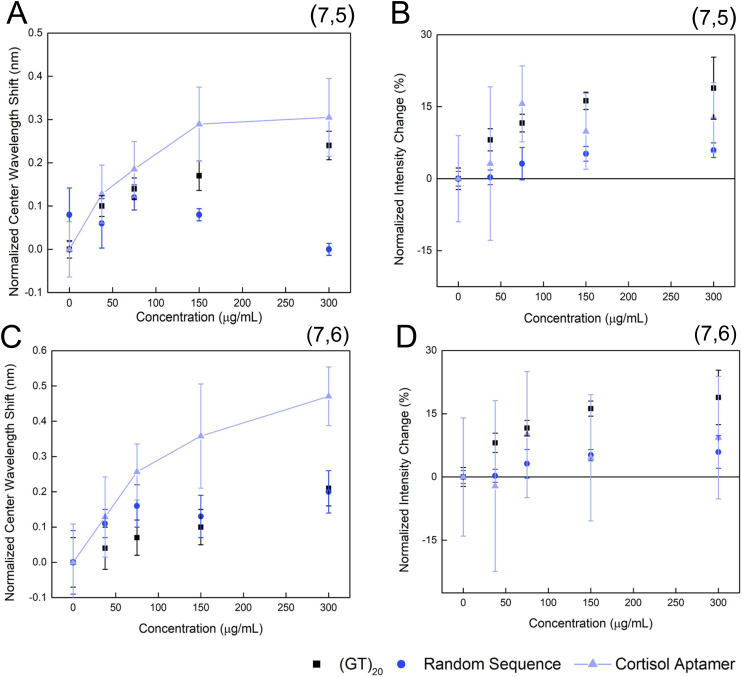
The cortisol aptamer induces a concentration-dependent response while non-specific ssDNA sequences do not. (A) Normalized center wavelength shift and (B) normalized intensity change, for the (7,5) chirality of HiPCO SWCNT functionalized with (GT)_20_, a cortisol-specific aptamer, and a 40-mer random sequence constructs. (C) Normalized center wavelength shift and (D) normalized intensity change, for the (7,6) HiPCO SWCNT functionalized with (GT)_20_, a cortisol-specific aptamer, and a 40-mer random sequence.

In this work, we developed a fluorescence nanosensor for cortisol using an aptameric biorecognition element. To evaluate whether other unspecific DNA sequences could also interact with cortisol, the response of SWCNT-(GT)_20_ and SWCNT-random 40mer sequence was evaluated. The (7,5) peak of SWCNT-(GT)_20_ (Fig. 3A) exhibited a minimal blue-shift consistent with linear non-specific adsorption. The SWCNT-random 40mer sequence construct also demonstrated very minor wavelength shifts in the (7,5) peak, with no clear correlation with cortisol concentration. For the (7,6) peak, both the SWCNT-(GT)_20_ and the SWCNT-random 40mer sequence constructs demonstrated a relatively minor concentration-dependent response (Fig. 3C), again consistent with linear non-specific adsorption to the surface of the nanotube most likely. The center wavelength shift response of these three constructs over the three hour period was also evaluated (Fig. S4 and S5), all exhibiting a sharp shift in the first 15 minutes. Neither control sequence demonstrated a significant intensity change at either chirality observed (Fig. 3B and D). A minor increase in intensity is observed for both chiralities with (GT)_20_, also likely consistent with non-specific adsorption to the surface of the carbon nanotube.

### The aptananosensor selectively detects cortisol and not estradiol

**Fig. 4:**
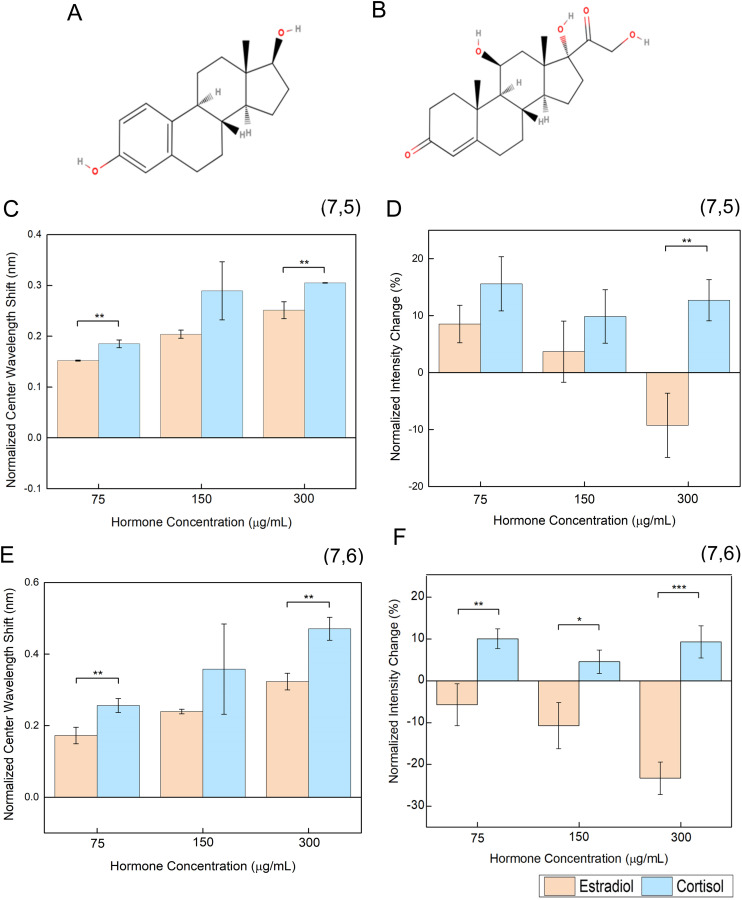
The aptananosensor selectively detects cortisol and not estrogen. Structures of (A) cortisol and (B) estradiol. (C) Normalized center wavelength shift and (D) intensity change for the (7,5) peak of HiPCO SWCNT-aptamer sensor. (E) Normalized center wavelength shift and (F) intensity change, for the (7,6) peak of HiPCO SWCNT-aptamer sensor. * = *p* < 0.05, ** = *p* < 0.01; *** = *p* < 0.001.

The selectivity of the sensor towards cortisol was evaluated by comparing the construct's response to estradiol. Estradiol, a form of estrogen, has structural similarities to cortisol (Fig. 4A and B) and both hormones are found in similar bodily fluids.^56^

The selectivity of center wavelength shifts for the (7,5) peak was statistically significant for cortisol compared to estrogen at the low and high tested concentrations, likely due to variability in the 150 μg mL^−1^ measurement (Fig. 4C), while the intensity change was selective only at 300 μg mL^−1^ (Fig. 4D). For the (7,6) peak, the center wavelength shift was selective at low and high concentrations, again due to variability in the middle test value (Fig. 4E). The intensity change, however, was significantly at all three concentrations tested (Fig. 4F). Notably, sensor center wavelength shifts in response to estradiol were in the same direction as cortisol, though slightly less. In contrast, the changes in intensity have a distinct opposite response for each hormone, which is more pronounced at higher concentrations. In comparison, the previous CoPhMoRe-SWCNT approach to cortisol detection found that the intensity-based response to estradiol was ∼50% of that compared to cortisol at ∼37 μg mL^−1^ of analyte.^39^ Potential improvements in selectivity of wavelength response may be made by screening passivation agents to coat the SWCNT surface to block any nonspecific interactions.^57^ This is further strengthened by prior molecular dynamics simulations with cortisol and the CoPhMoRe-SWCNT sensor, demonstrating cortisol may interact with the SWCNT surface within a polymeric binding pocket, thus indicating the necessity of directing interactions to the sensing element in the current design.^39^

### Sensor performance in aCSF and a-sweat

**Fig. 5:**
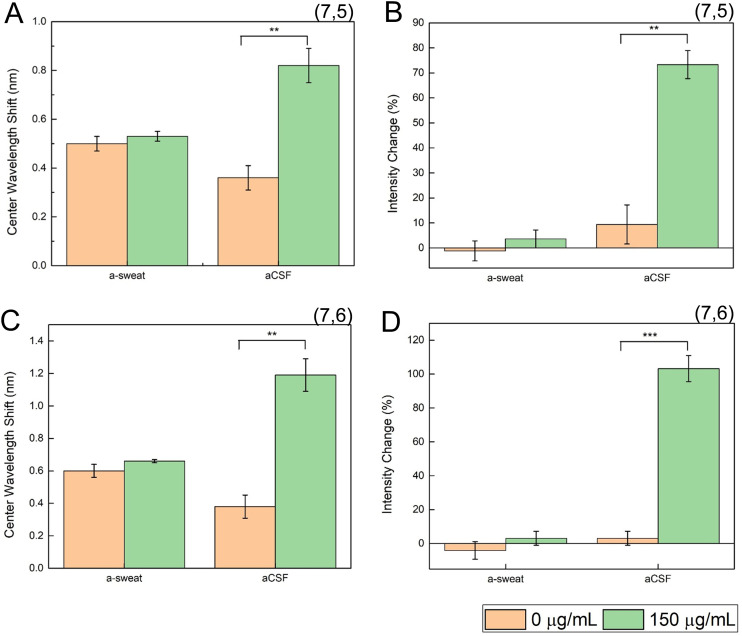
The aptananosensor is capable of detecting cortisol in aCSF. Center wavelength shift (A) and intensity change (B) for the (7,5) peak of HiPCO SWCNT-aptamer sensor, in a-sweat and aCSF. Center wavelength shift (C) and intensity change (D) for the (7,6) peak of HiPCO SWCNT-aptamer sensor, in a-sweat and aCSF. ** = *p* < 0.01, *** = *p* < 0.001.

To further evaluate the translational potential of this sensor, it was evaluated in 80% artificial sweat (a-sweat) and artificial cerebrospinal fluid (aCSF), which represent two potential complex biofluids in which elevated cortisol levels may be found in disease conditions. For example, cortisol levels are elevated in the CSF of patients with acute bacterial meningitis as well as cognitive impairment, which directly correlated with increased levels of inflammatory cytokines such as IL-6, TNF-α, and IL-1β, whereas decreased levels of cortisol are found in the CSF of patients with multiple sclerosis.^58–60^ Cortisol levels in the sweat are also directly linked to systemic stress levels.^61^ We found the aptananosensor demonstrated a significant wavelength and intensity change to 150 μg mL^−1^ cortisol in aCSF (Fig. 5). Neither peak, however, demonstrated a significant response to 150 μg mL^−1^ of cortisol in artificial sweat, potentially due to pH-induced aptamer conformational changes in sweat.^62^ While the previous cortisol SWCNT sensor was not tested in these biofluids, nor *in vivo*, the related progesterone sensor from the same study was embedded in a hydrogel and implanted into mice,^30^ wherein it was able to report the presence of progesterone.^39^

The biorecognition capabilities of an aptamer can be influenced by pH, either through protonation of the binding site or by partial denaturation of the aptamer structure, which may impact sensor performance in sweat samples.^63^ It is well-established that aptamer functionality is closely-related to the conditions in which it was selected, especially pH and salt concentrations,^64,65^ and thus it may be necessary to buffer pH if this aptamer is used, or to use a different aptamer sequence tailored to each condition. For pending clinical translation, it would be necessary to evaluate the stability of the aptamer, sensor construct, and signal in various buffers and over time.

## Conclusions

In this work, we engineered a SWCNT-aptamer based sensor for the stress hormone cortisol. The sensor demonstrated a center wavelength shift and intensity change in the presence of cortisol, whereas non-specific ssDNA sequences complexed with SWCNT did not. The sensor also demonstrated selective changes in intensity for cortisol compared to the similar molecule estradiol. Moreover, the sensor exhibited response to cortisol in aCSF, showing its potential use for clinical applications. Overall, the findings indicate that our engineered cortisol aptananosensor holds promise as a potential diagnostic tool. Compared to traditional methods, this approach offers the potential for *in vivo* monitoring and point-of-care detection, in a cost-effective cortisol manner.

## Conflicts of interest

There are no conflicts of interest to declare.

## Supplementary Material

SD-004-D5SD00085H-s001

## Data Availability

Supplementary information is available. See DOI: https://doi.org/10.1039/D5SD00085H. Raw and processed spectral data will be made available to requesters upon reasonable request. Matlab code used for data processing will also be made available to requesters upon reasonable request.
