# Supplementary material for: A near-infrared fluorescent aptananosensor enables selective detection of the stress hormone cortisol in artificial cerebrospinal fluid
Source: Sens Diagn. 2025 Oct 7;4(12):1103–13. doi: 10.1039/d5sd00085h (PMC12502572; doi:10.1039/d5sd00085h)
Supplement: SD-004-D5SD00085H-s001 [file SD-004-D5SD00085H-s001.pdf]

Supplementary Information (SI) for Sensors & Diagnostics.
This journal is © The Royal Society of Chemistry 2025

A near-infrared fluorescent aptananosensor enables selective detection of the stress hormone cortisol in artificial cerebrospinal fluid

Jessica Kretli Zanetti[1^], Maria Celina Stefoni[1,2^], Catarina Ferraz[1], Amelia Ryan[1], Atara Israel[1], and Ryan Williams[1,3*]

[1]The City College of New York, Biomedical Engineering, New York, NY 10031

[2] Departamento de Química Inorgánica, Analítica y Química Física, Facultad de Ciencias Exactas y Naturales (DQIAQF), Universidad de Buenos Aires, and Instituto de Química Física de los Materiales, Medio Ambiente y Energía (INQUIMAE), CONICET-UBA, Buenos Aires C1428, Argentina

[3]Stony Brook University, Department of Medicine, Division of Nephrology & Hypertension, Stony Brook, NY 11794

[^]Equal contribution

***ryan.williams@stonybrookmedicine.edu**

Supplementary Figures S1-S5

Supplementary Figures

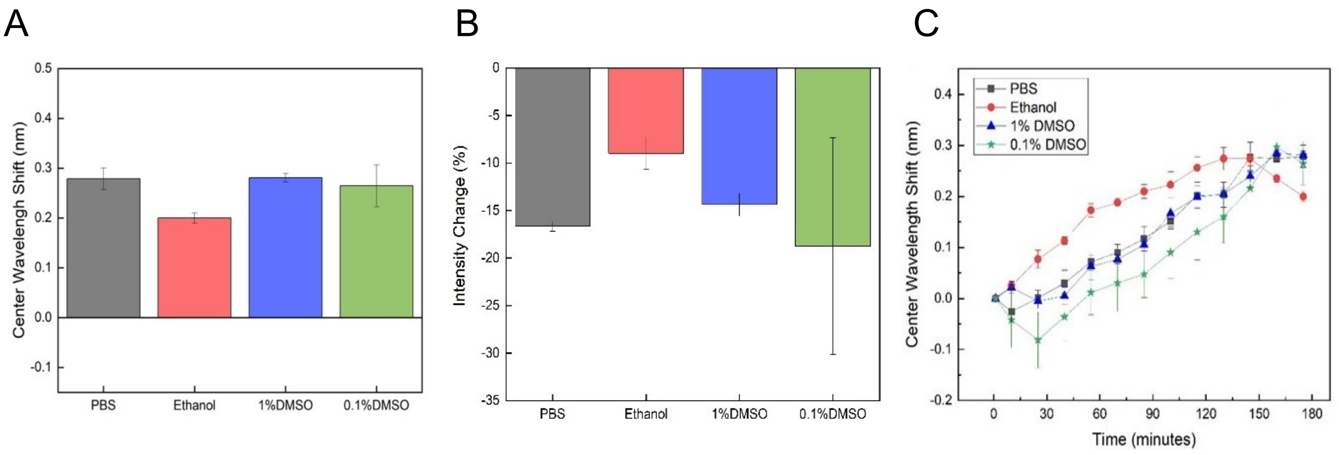

Figure S1: (A) Center wavelength shift and (B) intensity change, in HiPCo SWCNT (7,5) chirality in response to PBS, ethanol, 1% DMSO and 0.1% DMSO, after three hours (C) Center wavelength shift for the (7,5) peak during a 3-hour period, for different solvents.

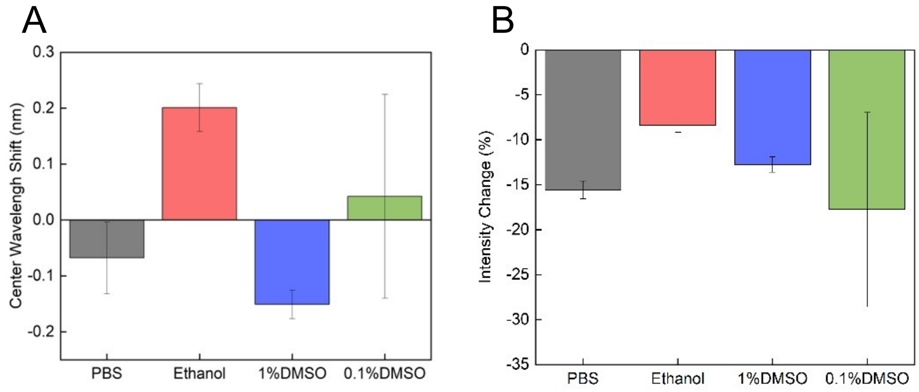

Figure S2: (A) Center wavelength shift and (B) intensity change in HiPCO SWCNT (7,6) chirality in response to PBS, ethanol, 1% DMSO and 0.1% DMSO after 3 hours.

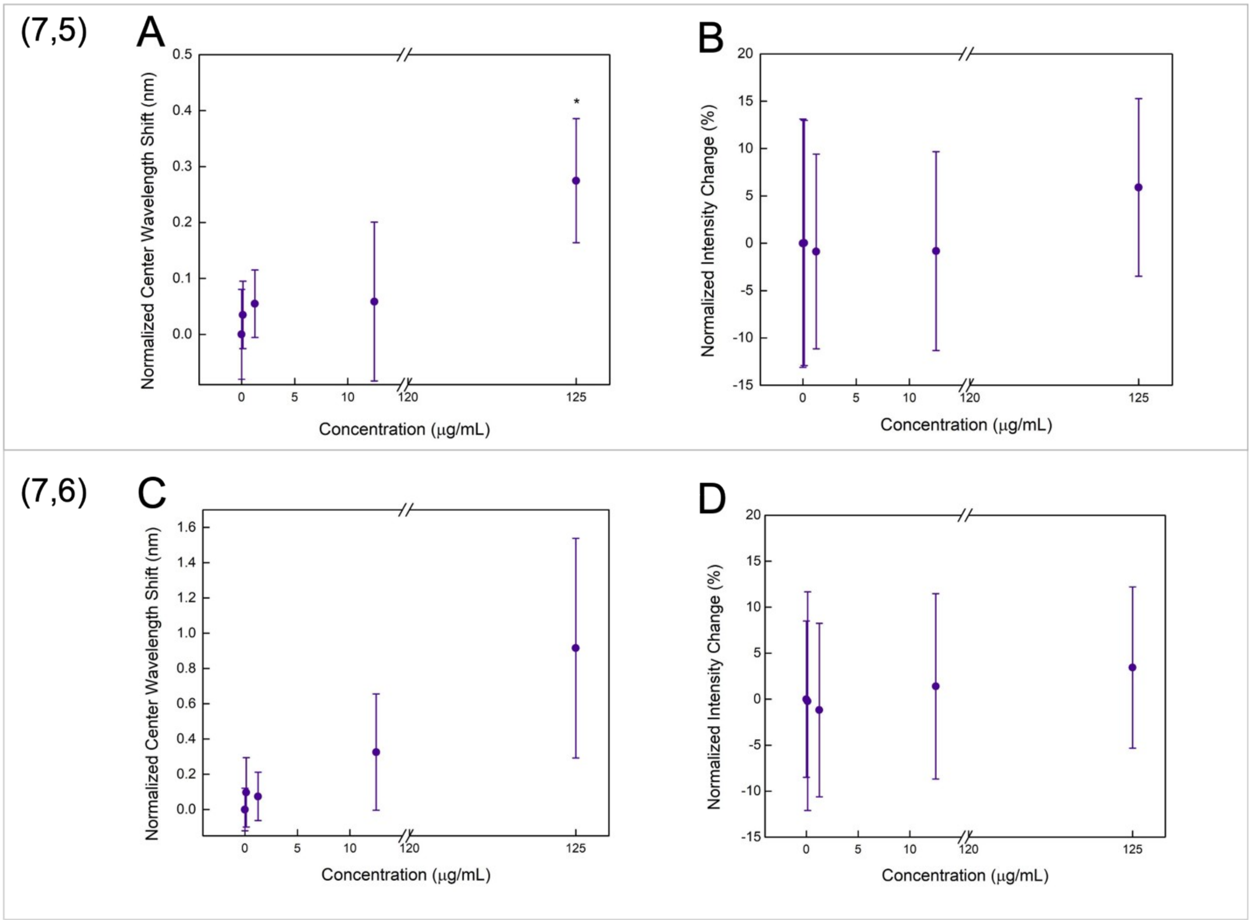

Figure S3: (A) Normalized center wavelength shift and (B) normalized intensity changes, for (7,5) chirality of (6,5)-enriched SWCNT formulation. (C) Normalized center wavelength shift and (D) normalized intensity changes, for (7,6) chirality of (6,5)-enriched SWCNT formulation.

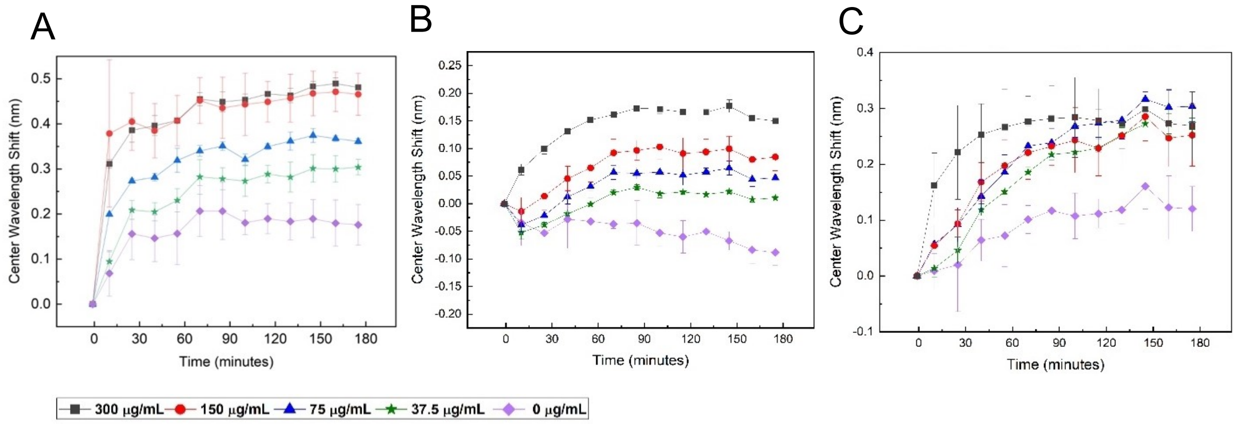

Figure S4: Center wavelength shift over a 3-hour period, for the (7,5) chirality of HiPCO SWCNT functionalized with (A) cortisol-specific aptamer (B) $(GT)_{20}$ and (C) a random 40-mer sequence.

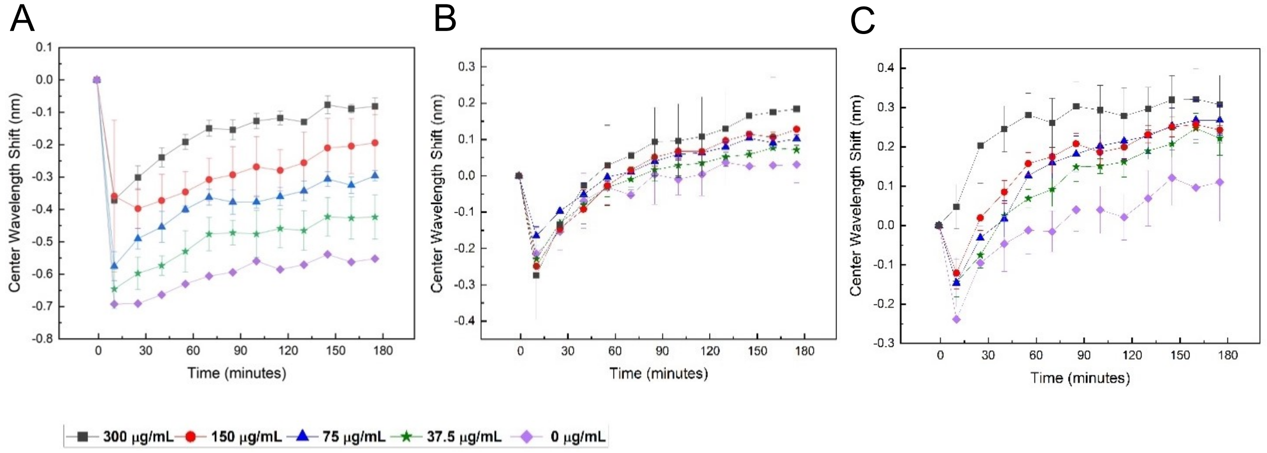

Figure S5: Center wavelength shift over a 3-hour period, for the (7,6) chirality of HiPCO SWCNT functionalized with (A) cortisol-specific aptamer (B) $(GT)_{20}$ and (C) a random 40-mer sequence.
